# Gut mucosal microbiota profiles linked to development of positional-specific human colorectal cancer

**DOI:** 10.3934/microbiol.2024035

**Published:** 2024-09-24

**Authors:** Chunze Zhang, Mingqian Ma, Zhenying Zhao, Zhiqiang Feng, Tianhao Chu, Yijia Wang, Jun Liu, Xuehua Wan

**Affiliations:** 1 Department of Colorectal Surgery, Tianjin Union Medical Center, Nankai University, Tianjin, China; 2 School of Integrative Medicine, Tianjin University of Traditional Chinese Medicine, Tianjin, China; 3 Tianjin institute of spinal surgery, Tianjin Union Medical Center, Nankai University, Tianjin, China; 4 Department of Radiology, The Fourth Central Hospital Affiliated to Nankai University, Tianjin, China; 5 TEDA Institute of Biological Sciences and Biotechnology, Nankai University, TEDA, Tianjin, China; 6 Tianjin Institute of Coloproctology, Tianjin, China

**Keywords:** gut microbiota, 16S rRNA gene, colorectal cancer, right-sided CRC, left-sided CRC, rectal CRC, biomarker

## Abstract

Colorectal cancer (CRC) continuously ranks as the third most common cause of cancer-related deaths worldwide. Based on anatomical classifications and clinical diagnoses, CRC is classified into right-sided, left-sided, and rectal CRC. Importantly, the three types of positional-specific CRC affect the prognosis outcomes, thus indicating that positional-specific treatments for CRC are required. Emerging evidence suggests that besides host genetic and epigenetic alterations, gut mucosal microbiota is linked to gut inflammation, CRC occurrence, and prognoses. However, gut mucosal microbiota associated with positional-specific CRC are poorly investigated. Here, we report the gut mucosal microbiota profiles associated with these three types of CRC. Our analysis showed that the unique composition and biodiversity of bacterial taxa are linked to positional-specific CRC. We found that a combination of bacterial taxa can serve as potential biomarkers to distinguish the three types of CRC. Further investigations of the physiological roles of bacteria associated with positional-specific CRC may help understand the mechanism of CRC progression in different anatomical locations under the impact of gut mucosal microbiota.

## 1. Introduction

Colorectal cancer (CRC) is one of the most common causes of cancer-related deaths, with the third highest mortality and the fourth highest incidence worldwide according to GLOBOCAN 2020 (global cancer statistics) [1]. Although accumulations of genetic and epigenetic alterations are well investigated for their roles in driving human CRC progression, emerging evidence shows that gut microbiota are pivotal factors that are strongly linked to gut inflammation and CRC initiation and progression [2],[3]. The development of bioinformatics analyses on the 16S ribosomal RNA (rRNA) gene and microbial metagenomes facilitates uncovering the composition and biodiversity of gut mucosal microbiota that are collected from patients with CRC. Using these sequencing technologies, more than 1013 microorganisms have been identified to colonize within the human gastrointestinal tract, of which the majority were bacteria [4],[5]. In total, 1057 microbial species, including bacteria (90.5%), archaea (0.8%), and eukarya (8.7%) have been identified within the human gastrointestinal tract [6]. Large-scale sequencing investigations have identified specific bacterial compositions that are associated with CRC initiation and progression [7]. In addition, certain bacterial species that are associated with CRC development, such as *Fusobacterium nucleatum*, *Peptostreptococcus anaerobius*, *Bacteroides fragilis*, and *Eubacterium rectale* have been studied for their pathogenic roles in driving CRC progression [8]–[11].

The human intestinal tract is formed from a network of multiple cell lineages, which have positional-specific embryonic origins [12]–[14]. The composition and complexity of intestinal cells dynamically change across the intestinal axis. According to anatomical classifications, CRC is mainly classified as right-sided CRC (that starts from cecum to ascending colon and then hepatic flexure), left-sided CRC (that starts from splenic flexure to descending colon and then sigmoid colon), and rectal CRC [15]. Various studies suggest that these three types of positional-specific CRC show clinically distinct differences in the prognosis and treatment outcomes [16]–[20]. Right-sided CRC has a worse prognosis than left-sided CRC, which may be caused by positional-specific compositions, functions of immune cell populations, and gut mucosal microbiota in the tumor microenvironment (TME). The difference of immune cell populations between left-sided and right-sided CRC has been investigated [15]. On the other hand, bacterial taxa associated with positional-specific CRC were also investigated in Japan and UK cohorts [21],[22]. Based on 16S rRNA amplicon sequencing, *Fusobacterium* was reported to be dominant in left-sided CRC (n = 37), whereas *Blautia*, *Eryspelotrichales*, *Holdemanella*, *Faecalibacterium*, *Subdoligranulum*, and *Dorea* were found to be the dominant intestinal microbiota in right-sided CRC (n = 16) [21]. Another study compared on-tumor microbiota to off-tumor microbiota. Species of *Lachnoclostridium*, *Selenomonas*, and *Ruminococcus* were enriched in right-sided CRC (n = 17), whereas *Methylophilaceae*, *Vadin* BE97, *Alloprevotella*, *Intestinibacter*, *Romboutsia*, and *Ruminococcus* were enriched in left-sided CRC (n = 7) [22]. However, the differences of the gut mucosal microbiomes in positional-specific CRC have not been fully understood yet, especially for a Chinese cohort.

In this study, to reveal the composition and biodiversity of gut mucosal microbiota associated with positional-specific CRC in the TME, we analyzed the gut mucosal microbiomes from 75 patients with CRC and 26 healthy controls. Our data revealed microbiome structures and bacterial taxa as potential biomarkers associated with positional-specific CRC, which may help understand positional-specific CRC occurrences and prognoses.

## 2. Materials and methods

### 2.1. Gut microbiota sample collection

The patients and healthy controls in Tianjin typically eat similar northern food, including wheat or rice, meat, and vegetables. Written informed consent was obtained from all the participants prior to their inclusion in the study. All the protocols and procedures were approved by the Medical Ethics Board of Tianjin Union Medical Center (2021B31). All the patients and healthy controls had bowel preparation via an oral polyethylene glycol-electrolyte. For each patient, samples were collected from three intestinal locations: the on-tumor (T) site, the adjacent-tumor (P) site, and the off-tumor (N) site. The average distance of collected samples between the on-tumor site and the adjacent-tumor site was around 2 cm. The average distance of collected samples between the on-tumor site and the off-tumor site was around 20 cm. A cotton swab was used to dip on the intestinal surface of the tissue. Samples of healthy controls were collected by colonoscopy (Olympus, Japan), which was implemented according to routine procedure. When the participants were diagnosed as healthy people, cotton swabs were used to dip on the surfaces of probes to collect samples for the healthy controls (H). Each sample was rinsed in 1 mL of physiological saline. Then, 200 µL of the solution was used for the bacterial DNA extraction. The clinicopathological features of the patients are summarized in Table 1.

**Table 1.** Information of 75 patients with CRC involved in the study. For tumor differentiation, ‘P’ represents poorly, ‘M’ represents moderately, and ‘W’ represents well differentiated carcinomas, respectively.

Position	Rectum	Left-Colon	Right-Colon
No.	43	21	11
Male/female	29/14	13/8	3/8
Age (mean, range)	63.9 (29–81)	62.7 (35–82)	62.9 (45–77)
Stages	I (4), II (20), III (12), IV (7)	I (2), II (7), III (9), IV (3)	I (2), II (3), III (4), IV (2)
Differentiation	P (9), MP (11), M (22), WM (1)	P (1), MP (6), M (12), WM (1), W (1)	P (2), MP (4), M (4), WM (1)

### 2.2. 16S rRNA amplicon preparation and sequencing

For the 16S rRNA amplicon preparation, bacterial genomic DNA was isolated using the ZR Fungal/Bacterial DNA kit (Zymo Research, Irvine, CA, USA). The amounts of bacterial genomic DNA were quantified using the Quant-iT PicoGreen dsDNA assay kit (Thermo Fisher, Sunnyvale, CA, USA). The 16S rRNA amplicon sequencing libraries targeting the V3-V4 region were prepared according to the Illumina manufactory manual. The amplification primers included a forward primer (5′TCGTCGGCAGCGTCAGATGTGTATAAGAGACAGCCTACGGGNGGCWGCAG) and a reverse primer (5′GTCTCGTGGGCTCGGAGATGTGTATAAGAGACAGGACTACHVGGGTATCTAATCC), according to the Illumina manufactory manual. The amplified DNA libraries were purified using AMPure XP beads (Beckman Coulter, Fullerton, CA, USA). The amounts of libraries were quantified using the Quant-iT PicoGreen dsDNA assay kit (Thermo Fisher, Sunnyvale, CA, USA). The amplicon libraries were bidirectionally sequenced (2 × 300 bp) on the Illumina MiSeq platform.

### 2.3. OTU picking and analysis of 16S rRNA amplicons

Quality control and filtering of the raw sequencing reads were performed using FastQC (https://www.bioinformatics.babraham.ac.uk/projects/fastqc/). The filtered paired-end reads were assembled using PandaSeq, v2.10 [23], with default parameters. De novo Operational Taxonomic Unit (OTU) picking, a taxonomic assignment, and a diversity analysis were carried out using QIIME, v1.9.1, with the Greengenes database, v13.8 (http://qiime.org/home_static/dataFiles.html) [24]. In brief, the assembled sequences were clustered against one another without an external reference sequence, and de novo OTUs were picked using a similarity threshold of 97%, which is commonly used to define bacterial species. Chimera detection and filtering were performed using USEARCH, version 6.1. Next, a taxonomy was assigned to the OTU representative sequences. The 16S rRNA sequencing reads were submitted to the National Center for Biotechnology Information (NCBI) Sequence Read Archive (SRA) database under the accession number PRJNA606879.

### 2.4. Determination of bacterial species associated with positional-specific CRC

The compositional and structure differences of gut mucosal microbiota in different positional-specific CRC and tissue-specific sites were initially analyzed using a principal component analysis (PCA). The PCA was performed using R, v4.0.3, with the factoextra package, v1.0.7 [25]. The confidence ellipse type of the PCA was set to Euclid, and the confidence level of the PCA was set to 95%. The alpha diversity, bacterial taxa at the Phylum level, and the linear discriminant analysis (LDA) effect size (LEfSe) were analyzed using the online MicrobiomeAnalyst software (https://dev.microbiomeanalyst.ca/MicrobiomeAnalyst/home.xhtml) [26],[27]. For multi tests, an adjusted P < 0.05 was considered significant. To perform the LEfSe analysis, the cutoff threshold for the FDR-adjusted P-value was set as 0.05, and the cutoff threshold for the LDA score was set to 3.0. To obtain the proportion value, the OTU number of each bacterial taxon was divided by the total OTU number of the sample. The genera with proportions >0.1% identified at T-sites of right-sided, left-sided, and rectal CRC were analyzed for a Venn diagram [28]. The change trends of the microbial proportions in conditions at the on-tumor sites were used to classify the bacterial taxa to four groups: group I, right-sided CRC (trc) > left-sided CRC (tlc) > rectal CRC (tr); group II, right-sided CRC (trc) > left-sided CRC (tlc) < rectal CRC (tr); group III, right-sided CRC (trc) < left-sided CRC (tlc) > rectal CRC (tr); and group IV, right-sided CRC (trc) < left-sided CRC (tlc) < rectal CRC (tr)). A heatmap was visualized using the R command heatmap. Specific genera were selected to compare their proportions at the three sites (N-, P- and T-sites) among positional-specific CRC. To identify the bacterial group associated with right-sided CRC, the proportions of bacterial taxa at the on-tumor site should be higher than those at the off-tumor site and the adjacent-tumor site. Meanwhile, after those proportions of bacterial taxa at the on-tumor sites were normalized by proportions at the off-tumor sites, the normalized values of bacterial taxa in right-sided CRC should be higher than those in left-sided and rectal CRC. Bacterial groups associated with left-sided CRC and rectal CRC were selected in a similar manner. Bacterial taxa that showed the highest fold changes of the average proportions between the indicated conditions were selected to test for potential biomarkers. Receiver operating characteristic (ROC) curves were analyzed using the GraphPad Prism program, v6.01. The area under the ROC curve (AUC) metrics and P values were calculated. A P < 0.05 was considered significant.

### 2.5. Statistical analysis

Statistical analyses were performed using GraphPad Prism, v6.01. The statistical significances of two sample comparisons were calculated using the student's *t* test. The statistical significances of multiple sample comparisons were calculated using a one-way analysis of variance (ANOVA) with the Kruskal–Wallis test. *, p < 0.05.

## 3. Results

### 3.1. Clinicopathological features of patients with CRC

The gut mucosal microbiota samples were collected from 43 patients clinically diagnosed with rectal CRC, 21 patients with left-sided CRC, 11 patients with right-sided CRC, and 26 healthy controls, in Tianjin Union Medical Center, China. The patients' clinicopathological data are listed in Table 1. The average ages of patients with right-sided, left-sided, and rectal CRC were similar (Table 1). The majority of carcinomas were not well differentiated (Table 1). The stage distributions of patients with right-sided, left-sided, and rectal CRC were similar, and most were stages II and III (Table 1).

### 3.2. Altered alpha-diversity of gut mucosal microbiota among positional-specific CRC at adjacent-tumor site

**Figure 1.**
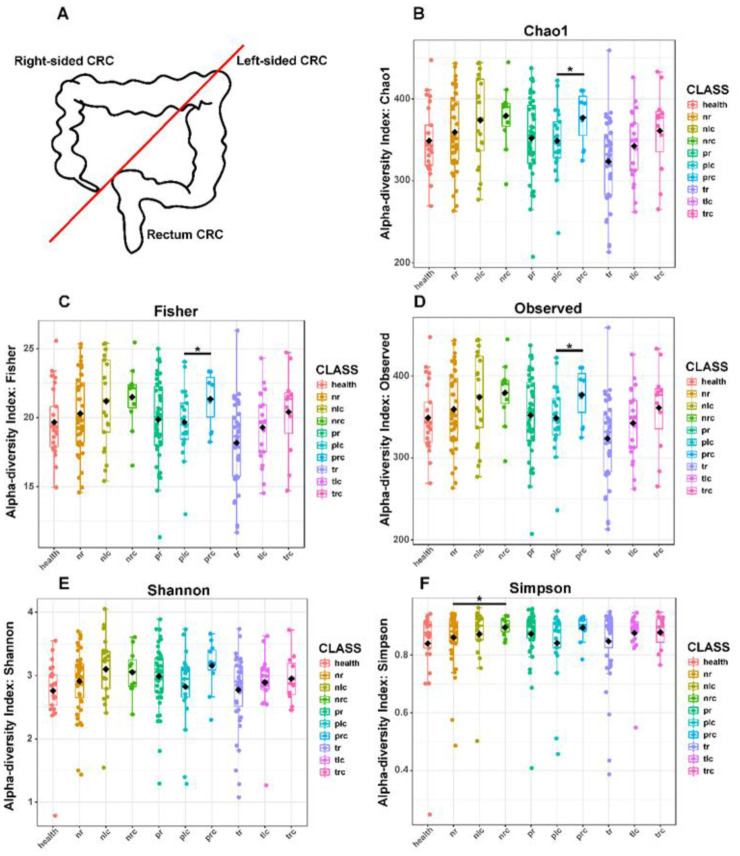
Microbial alpha-diversities showing differences at the on-tumor (T), adjacent-tumor (P), and off-tumor (N) sites of patients with right-sided, left-sided, and rectal CRC. (A) Schematic diagram showing anatomical positions of right-sided, left-sided, and rectal CRC. (B) Alpha diversity evaluated using the Chao1 index. (C) Alpha diversity evaluated using the Fisher index. (D) Alpha diversity evaluated using the Observed OTU index. (E) Alpha diversity evaluated using the Shannon diversity index. (F) Alpha diversity evaluated using the Simpson index. Health: healthy controls; nr: off-tumor site of patient with rectal CRC; nlc: off-tumor site of patient with left-sided CRC; nrc: off-tumor site of patient with right-sided CRC; pr: adjacent-tumor site of patient with rectal CRC; plc: adjacent-tumor site of patient with left-sided CRC; prc: adjacent-tumor site of patient with right-sided CRC; tr: on-tumor site of patient with rectal CRC; tlc: on-tumor site of patient with left-sided CRC; trc: on-tumor site of patient with right-sided CRC. The alpha-diversity differences were compared using a one-way ANOVA with the Kruskal–Wallis test. *, p < 0.05. Nr, nlc, and nrc were compared between each other. Pr, plc, and prc were compared between each other. Tr, tlc, and trc were compared between each other. Only p < 0.05 was shown in the figures.

To evaluate the structure variation of microbiota among different locations within the intestinal tract (Figure 1A), we assessed the alpha diversities of microbiota in biopsy samples collected from the on-tumor (T), adjacent-tumor (P), and off-tumor (N) sites of patients with the three types of CRC. The 16S rRNA gene hypervariable V3-V4 regions were sequenced and then analyzed for five alpha-diversity indices: Chao1, Fisher, Observed OTUs, Shannon, and Simpson. An analysis of these alpha-diversity indices showed that, based on the Chao1, Fisher and Observed OTUs indices, the alpha-diversities of microbiota between right-sided (prc) and left-sided (plc) CRC at the adjacent-tumor (P) site were significantly different (P < 0.05) (Figure 1B–D). Based on the Simpson index, the alpha diversities between right-sided (nrc) and rectal (nr) CRC at the off-tumor (N) sites showed a significant difference (P < 0.05) (Figure 1F). Although the mean values of the alpha diversities of microbiota in right-sided (trc) CRC were greater than those in left-sided (tlc) and rectal (tr) CRC at the on-tumor (T) site in most cases, they were not significantly different (P > 0.05) (Figure 1B–F). Consistently, previous work reported that the alpha diversity indices showed no significant difference between right-sided and left-sided CRC at the on-tumor (T) site [22].

### 3.3. Altered beta-diversity of gut mucosal microbiota among positional-specific CRC

**Figure 2.**
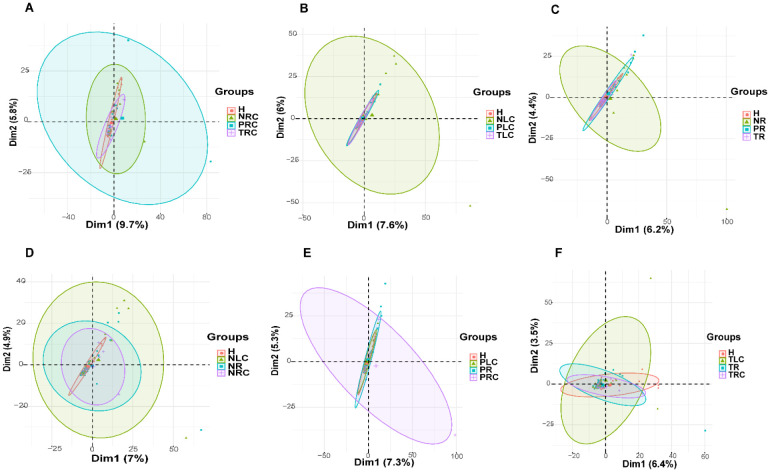
PCA showing microbiome profile differences among the on- (T), adjacent- (P), and off-tumor (N) sites of patients diagnosed with right-sided, left-sided, and rectal CRC. (A–C) PCA showing microbial diversities at the on-, off-, and adjacent-tumor sites of patients with right-sided (A), left-sided (B), or rectal (C) CRC. (D–F) PCA showing microbial diversities at the off- (D), adjacent- (E), or on- (F) tumor sites of patients with right-sided, left-sided, and rectal CRC. H: healthy controls; NR: off-tumor site of patient with rectal CRC; NLC: off-tumor site of patient with left-sided CRC; NRC: off-tumor site of patient with right-sided CRC; PR: adjacent-tumor site of patient with rectal CRC; PLC: adjacent-tumor site of patient with left-sided CRC; PRC: adjacent-tumor site of patient with right-sided CRC; TR: on-tumor site of patient with rectal CRC; TLC: on-tumor site of patient with left-sided CRC; TRC: on-tumor site of patient with right-sided CRC.

To compare the compositional differences of the microbiota structure diversities (beta-diversity) among positional-specific CRC, we carried out a PCA of the bacterial genera proportions identified at the T-, P-, and N-sites of patients with right-sided, left-sided, and rectal CRC. The microbiota structure diversities of the N-, P-, and T-sites showed differences (confidence ellipse radii and angel of PCA) for right-sided (nrc, prc and trc) and rectal (nr, pr and tr) CRC (Figure 2A, C). For left-sided CRC, only the N-site (nlc) showed differences (confidence ellipse radii and angel of PCA) from the P-sites (plc), T-sites (tlc), and healthy controls (H) (Figure 2B). Moreover, at each of the N- (nrc, nlc and nr), P- (prc, plc and pr), and T-sites (trc, tlc and tr), the microbiota structure diversities of right-sided, left-sided, and rectal CRC showed differences (confidence ellipse radii and angel of PCA) (Figure 2D–F). Thus, the microbial compositions and structure diversities vary among right-sided, left-sided, and rectal CRC, thus suggesting that specific bacterial genera may be associated with the three types of positional-specific CRC, most likely due to TME differences.

### 3.4. Gut mucosal microbiota profiles linked to positional-specific CRC

**Figure 3.**
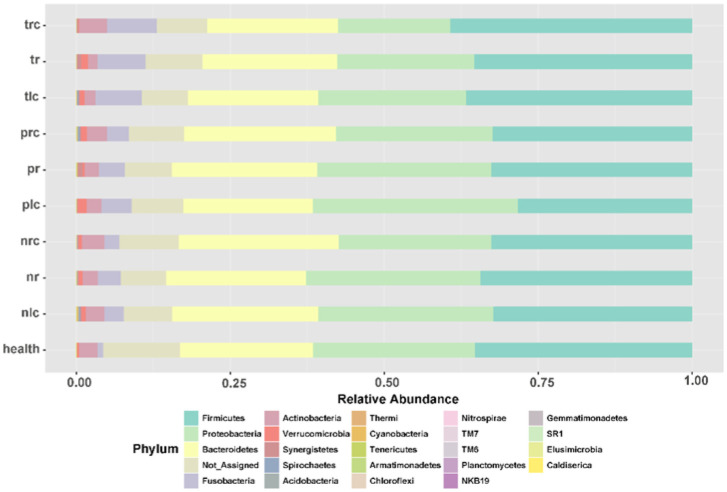
Comparison of bacterial phylum at the on-, off-, and adjacent-tumor sites of patients with right-sided, left-sided, and rectal CRC. Health: healthy controls; nr: off-tumor site of patient with rectal CRC; nlc: off-tumor site of patient with left-sided CRC; nrc: off-tumor site of patient with right-sided CRC; pr: adjacent-tumor site of patient with rectal CRC; plc: adjacent-tumor site of patient with left-sided CRC; prc: adjacent-tumor site of patient with right-sided CRC; tr: on-tumor site of patient with rectal CRC; tlc: on-tumor site of patient with left-sided CRC; trc: on-tumor site of patient with right-sided CRC. The conditions were not compared for a statistical analysis.

To show differences of the bacterial proportions in positional-specific CRC, we investigated the proportion differences of gut mucosal microbiota in right-sided, left-sided, and rectal CRC (Figure 3). For the data presented in Figure 3, the conditions were not compared for a statistical analysis. The most abundant and dominant phyla across all the conditions included *Firmicutes*, *Proteobacteria*, *Bacteroidetes*, and *Fusobacteria* (Figure 3). The proportions of *Fusobacteria* at the T-sites were higher than those at the N-sites for all the three types of positional-specific CRC, although the comparisons lacked a statistical significance (P > 0.05) (Figure 3). The proportions of *Bacteroidetes* and *Proteobacteria* were similar in right-sided, left-sided, and rectal CRC at the T-, P-, and N-sites (P > 0.05) (Figure 3).

**Figure 4.**
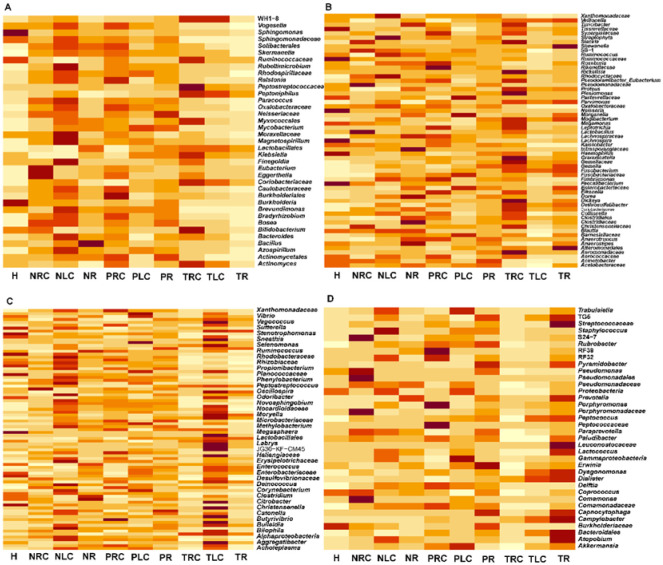
Heatmap comparisons of bacterial taxa proportions at the on-, off-, and adjacent-tumor sites of patients with right-sided, left-sided, and rectal CRC. (A) At the on-tumor sites, the bacterial proportions of right-sided CRC > left-sided CRC > rectal CRC. (B) At the on-tumor sites, the bacterial proportions of right-sided CRC > left-sided CRC < rectal CRC. (C) At the on-tumor sites, the bacterial proportions of right-sided CRC < left-sided CRC > rectal CRC. (D) At the on-tumor sites, the bacterial proportions of right-sided CRC < left-sided CRC < rectal CRC. H: healthy controls; NR: off-tumor site of patient with rectal CRC; NLC: off-tumor site of patient with left-sided CRC; NRC: off-tumor site of patient with right-sided CRC; PR: adjacent-tumor site of patient with rectal CRC; PLC: adjacent-tumor site of patient with left-sided CRC; PRC: adjacent-tumor site of patient with right-sided CRC; TR: on-tumor site of patient with rectal CRC; TLC: on-tumor site of patient with left-sided CRC; TRC: on-tumor site of patient with right-sided CRC. The highly changed conditions were visualized via a heatmap. The conditions were not compared for a statistical analysis.

Next, we compared the proportion differences of the gut mucosal microbiota at the T-, P-, and N-sites of patients with right-sided, left-sided, and rectal CRC. According to the proportion differences of bacterial genera and families at the T-sites of patients with right-sided, left-sided, and rectal CRC, the bacterial genera and families were classified into four groups: group I, right-sided CRC (trc) > left-sided CRC (tlc) > rectal CRC (tr); group II, right-sided CRC (trc) > left-sided CRC (tlc) < rectal CRC (tr); group III, right-sided CRC (trc) < left-sided CRC (tlc) > rectal CRC (tr); and group IV, right-sided CRC (trc) < left-sided CRC (tlc) < rectal CRC (tr)) (Figure 4). For the data presented in Figure 4, the conditions were not compared for a statistical analysis. In group I, bacterial genera such as *Bifidobacterium* showed the highest average proportion at the T-sites of patients with right-sided CRC (P < 0.05), whereas *Peptoniphilus*, *Klebsiella*, and *Actinomyces* showed the highest average proportions at the T-sites of patients with right-sided CRC, but with no statistical significances (P > 0.05) (Figure 4A). In group III, bacterial genera such as *Vagococcus*, *Selenomonas*, *Oscillospira*, *Moryella*, *Labrys*, *Citrobacter*, and *Aggregatibacter* showed the highest average proportions at the T-sites of patients with left-sided CRC, but with no statistical significances (P > 0.05) (Figure 4C). In group IV, bacterial genera such as *Streptococcaceae*, *Prevotella*, *Peptococcus*, *Dysgonomonas*, *Dialister*, *Campylobacter*, and *Atopobium* showed the highest average proportions at the T-sites of patients with rectal CRC, but with no statistical significances (P > 0.05) (Figure 4D). These data indicate that specific bacterial genera were enriched in positional-specific CRC, thus supporting the speculation that TME in right-sided, left-sided, and rectal CRC contain different profiles of gut mucosal microbiota.

### 3.5. Top abundant bacteria showing differences among right-sided/left-sided/rectal CRC

**Figure 5.**
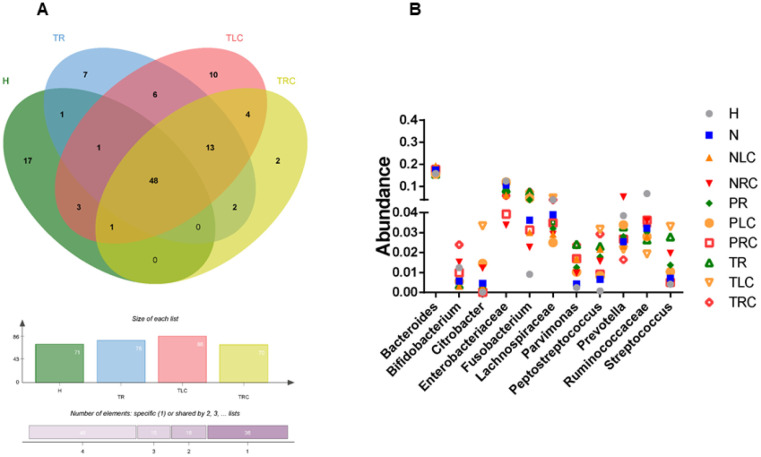
Comparison of the top abundant genera in different conditions. (A) Venn diagram visualizing a comparison of the genera with proportions > 0.1% at the on-tumor sites of patients with positional-specific CRC and healthy controls. (B) Comparison of the top 10 abundant genera under the conditions. H: healthy controls; NR: off-tumor site of patient with rectal CRC; NLC: off-tumor site of patient with left-sided CRC; NRC: off-tumor site of patient with right-sided CRC; PR: adjacent-tumor site of patient with rectal CRC; PLC: adjacent-tumor site of patient with left-sided CRC; PRC: adjacent-tumor site of patient with right-sided CRC; TR: on-tumor site of patient with rectal CRC; TLC: on-tumor site of patient with left-sided CRC; TRC: on-tumor site of patient with right-sided CRC. The conditions in Figure 5B were not compared for a statistical analysis.

To investigate the compositional differences of bacterial taxa among the patients with right-sided, left-sided, and rectal CRC, we selected and compared the bacterial taxa with high proportions (> 0.1%) at the T-sites of patients with the positional-specific CRC and healthy controls. The majority (48) of bacterial taxa were shared among the four conditions, whereas 2, 10, 7, and 17 bacterial taxa were unique to the on-tumor sites of patients with right-sided, left-sided, rectal CRC, and healthy controls, respectively (Figure 5A). *Pseudoramibacter_Eubacterium* and *Eikenella* were unique to the on-tumor sites of patients with right-sided CRC. *Sneathia*, *Treponema*, *Butyricimonas*, *Acidaminococcus*, *Catenibacterium*, *Desulfovibrio*, *Prevotellaceae*, *Agrobacterium*, *Xanthomonadaceae*, and *Veillonellaceae* were unique to the on-tumor sites of patients with left-sided CRC. *Shewanella*, *Morganella*, *Prevotella*, *Capnocytophaga*, *Pyramidobacter*, *Leuconostocaceae*, and *Comamonadaceae* were unique to the on-tumor sites of patients with rectal CRC. Again, these data indicate that different compositions and structures of bacterial taxa were enriched at the on-tumor sites of patients with right-sided, left-sided, and rectal CRC.

Next, we compared the top abundant bacterial genera/families among all ten different conditions (Figure 5B). For the data presented in Figure 5B, the conditions were not compared for a statistical analysis. A total of eleven bacterial genera/families, including *Bacteroides*, *Bifidobacterium*, *Citrobacter*, *Enterobacteriaceae*, *Fusobacterium*, *Lachnospiraceae*, *Parvimonas*, *Peptostreptococcus*, *Prevotella*, *Ruminococcaceae*, and *Streptococcus*, were found to be dominant (top abundant bacterial taxa) in one of the conditions. *Bacteroides* showed similar proportion levels in all the conditions, with no significant difference (P > 0.05). *Ruminococcaceae* was the most abundant family in the healthy controls compared to the other conditions (P < 0.05), whereas the proportion of *Enterobacteriaceae* showed in the healthy control was higher than those at the adjacent- and off-tumor sites of patients with right-sided CRC, although the comparisons lacked a statistical significance (P > 0.05). The proportion of *Prevotella* at the off-tumor sites of patients with right-sided CRC was higher than those in the other conditions, but with no statistical significance (P > 0.05). The proportion of *Bifidobacterium* was higher at the on-tumor sites of patients with right-sided CRC than those in the other conditions, but with no statistical significance (P > 0.05). The proportions of *Fusobacterium* and *Parvimonas* were higher at the on-tumor sites of patients with rectal CRC than those in other conditions, but with no statistical significance (P > 0.05). These data consistently suggest that different bacterial taxa with top proportions were enriched in the TME of right-sided, left-sided, and rectal CRC.

### 3.6. Altered gut mucosal microbiota signatures in positional-specific CRC

**Figure 6.**
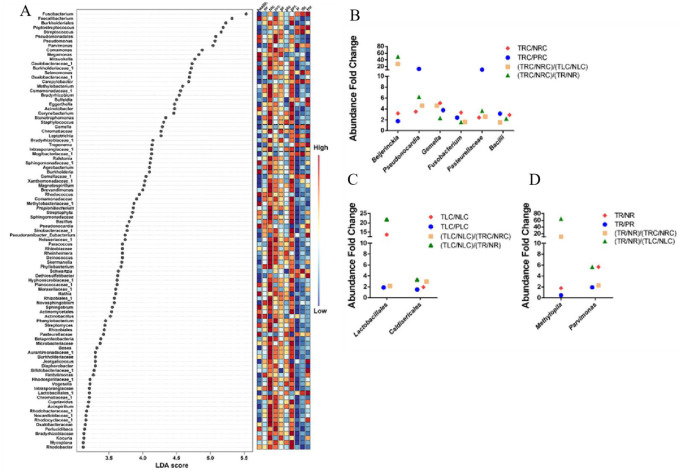
LEfSe comparison of bacterial taxa under different conditions. (A) Bacterial taxa with top LEfSe scores. (B) Bacterial taxa enriched at the on-tumor sites of patients with right-sided CRC. (C) Bacterial taxa enriched at the on-tumor sites of patients with left-sided CRC. (D) Bacterial taxa enriched at the on-tumor sites of patients with rectal CRC. Health: healthy controls; NR: off-tumor site of patient with rectal CRC; NLC: off-tumor site of patient with left-sided CRC; NRC: off-tumor site of patient with right-sided CRC; PR: adjacent-tumor site of patient with rectal CRC; PLC: adjacent-tumor site of patient with left-sided CRC; PRC: adjacent-tumor site of patient with right-sided CRC; TR: on-tumor site of patient with rectal CRC; TLC: on-tumor site of patient with left-sided CRC; TRC: on-tumor site of patient with right-sided CRC. All the data had statistical significances. P < 0.05 was considered significant.

The above data suggested that right-sided, left-sided, and rectal CRC were associated with distinct bacterial taxa. To predict potential key biomarkers that are associated with positional-specific CRC and may be used to distinguish positional-specific TME, we carried out the LEfSe method for this analysis. The top 100 bacterial taxa (LDA score > 3, P < 0.05) identified by the LEfSe method were shown in Figure 6A. Among them, *Fusobacterium* showed the highest LDA score (LDA score > 5.5), followed by *Faecalibacterium*, *Burkholderiales*, *Peptostreptococcus*, *Streptococcus*, *Pseudomonadales*, *Pseudomonas*, and *Parvimonas* (LDA score > 5) (Figure 6A). Six bacterial taxa, including *Beijerinckia*, *Pseudonocardia*, *Gemella*, *Fusobacterium*, *Pasteurellaceae*, and *Bacilli*, were enriched at the on-tumor sites of patients with right-sided CRC (Figure 6B). *Lactobacillales* and *Caldisericales* were enriched at the on-tumor sites of patients with left-sided CRC (Figure 6C). *Methylopila* and *Parvimonas* were enriched at the on-tumor sites of patients with rectal CRC (Figure 6D).

### 3.7. Bacterial taxa as potential biomarkers to distinguish right-sided/left-sided/rectal CRC

**Figure 7.**
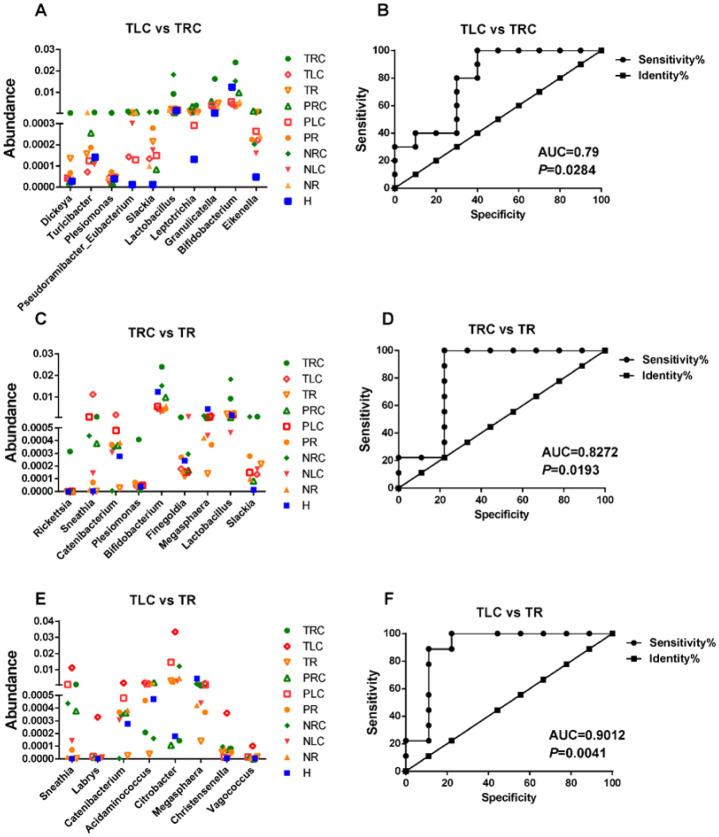
Identification of bacterial taxa as potential biomarkers to differentiate different positional-specific CRC. (A, C, E) Proportion comparisons of bacterial taxa as potential biomarkers to differentiate TLC from TRC (A), TRC from TR (C), or TLC from TR (E). (B, D, F) ROC curve analysis showing the diagnostic performance of the potential biomarkers to differentiate TLC from TRC (B), TRC from TR (D), and TLC from TR (F). H: healthy controls; NR: off-tumor site of patient with rectal CRC; NLC: off-tumor site of patient with left-sided CRC; NRC: off-tumor site of patient with right-sided CRC; PR: adjacent-tumor site of patient with rectal CRC; PLC: adjacent-tumor site of patient with left-sided CRC; PRC: adjacent-tumor site of patient with right-sided CRC; TR: on-tumor site of patient with rectal CRC; TLC: on-tumor site of patient with left-sided CRC; TRC: on-tumor site of patient with right-sided CRC. AUC: areas under the receiver-operating curve. P < 0.05 was considered significant. The conditions in Figure 7A, C, and E were not compared for statistical analyses.

To distinguish microbiota among right-sided, left-sided, and rectal CRC, the bacterial taxa with high proportions were specifically selected. *Dickeya*, *Turicibacter*, *Plesiomonas*, *Eubacterium*, *Slackia*, *Lactobacillus*, *Leptotrichia*, *Granulicatella*, *Bifidobacterium*, and *Eikenella* can serve as potential biomarkers for right-sided CRC to be distinguished from left-sided CRC (AUC = 0.79, P < 0.05) (Figure 7A, B). *Rickettsia*, *Sneathia*, *Catenibacterium*, *Plesiomonas*, *Bifidobacterium*, *Finegoldia*, *Megasphaera*, *Lactobacillus*, and *Slackia* can serve as potential biomarkers for right-sided CRC to be distinguished from rectal CRC (AUC = 0.83, P < 0.05) (Figure 7C and D). *Sneathia*, *Labrys*, *Catenibacterium*, *Acidaminococcus*, *Citrobacter*, *Megasphaera*, *Christensenella*, and *Vagococcus* can serve as potential biomarkers to distinguish left-sided CRC from rectal CRC (AUC = 0.90, P < 0.05) (Figure 7E, F).

## 4. Discussion

Human right and left colons develop from two different embryological origins [14],[29]. The primitive midgut develops into the ascending and proximal transverse colons, which form the right colon. The primitive hindgut develops into the distal transverse, descending, and sigmoid colons, which constitute the left colon. Additionally, the latter develops into rectum that is located at the end of the digestive tract. After the development process, the right and left colons are supplied by different blood vessels (right colon: superior mesenteric artery; left colon: inferior mesenteric artery) and regulated by different innervations (right colon: sympathetic innervation; left colon: parasympathetic innervation). The difference of anatomical locations where tumor can occur leads to complex morphologies, molecular characteristics, and histology of right-sided, left-sided, and rectal CRC [30]. Moreover, a comparison of right-sided and left-sided CRC by single-cell transcriptome profiling suggests that compositional structures of residential intestinal cells and migratory immune cells dramatically vary, while the expression levels of signature marker genes differ between right-sided and left-sided CRC [15]. The heterogeneity of TME makes CRC a complicated disease and affects its prognosis through multiple factors.

The interplay between gut mucosal microbiota and colon cells leads to heterogeneous TME and CRC development. The complex heterogeneity of TMEs in right-sided and left-sided CRC may harbor distinct compositional structures of the gut mucosal microbiota, which, in turn, impels carcinogenesis in a positional-specific manner. Two studies reported bacterial taxa specific to right-sided and left-sided CRC in Japanese and British cohorts, respectively [21],[22]. In this work, we identified the bacterial taxa and potential biomarkers specific to right-sided and left-sided CRC in a Chinese cohort, which were dramatically different from those found in CRC samples collected from the Japanese (fecal samples) and British (mucosal tissue) patients. These discrepancy results may be due to the geographical and ethnicity-specific heterogeneity of the TME and gut microbiota. On the other hand, our results consistently support that the compositional structures and biodiversities of gut mucosal microbiota are anatomically specific to the CRC locations, which may affect the prognosis outcomes of positional-specific CRC.

In this work, we compared the compositional structures and biodiversities of gut mucosal microbiota at the on-tumor, adjacent-tumor, and off-tumor sites of patients diagnosed with right-sided, left-sided, and rectal CRC. Our alpha-diversity analysis suggests that the alpha-diversity indices of right-sided and left-sided CRC were significantly different (P < 0.05) at the adjacent-tumor (P) site but not on-tumor (T) site, which is consistent with a previous report for a European cohort [22]. These data indicate that the ecosystem of the on-tumor sites may be relatively consistent among the three anatomically classified organ sections, thus limiting the species numbers within these samples. In contrast, at the adjacent-tumor sites, the impact of the original ecosystem of the anatomical location may show a stronger effect than those of the tumors, thus leading to a significant difference in the alpha diversity. Although the P values were above 0.05 and indicated no statistical significance, the comparisons of the mean values of the alpha diversity indices suggest that the bacterial biodiversity decreased from the off-tumor site to the adjacent-tumor site and then the on-tumor site. It is possible that fewer bacterial species are able to thrive at the on-tumor site because dominant bacteria such as *Fusobacteria* may take up most of the nutrients, and the non-pathogenic bacteria are unable to evade the immune surveillance system of the host. In addition, our data suggest that the bacterial biodiversity decreased from right-sided CRC to left-sided CRC and then rectal CRC at the on-tumor, adjacent-tumor, and off-tumor sites. These data indicate that the anatomical location is a strong impact factor for the pathogenic bacterial activities in promoting CRC, which should be carefully considered for gut microbiota profiling and prognosis improvements.

The PCA, LefSe, and ROC curve analyses consistently demonstrated that the compositional structures of gut mucosal microbiota dramatically varied among the three anatomical organ sections. Although both the left-sided colon and rectum originate from the hindgut, the compositional structures of gut mucosal microbiota in these two anatomical organ sections show distinct differences. To distinguish the tumor sites between the anatomical locations, signature combinations of specific bacterial taxa were selected and can serve as potential biomarkers to predict the positional-specific CRC with high AUC values. For some comparisons, the calculated P values were above 0.05, which suggested no statistical significances for the differences. P values can be affected by many factors such as the sample size. In future works, testing the results in large-scale positional-specific CRC samples may help resolve this problem and provide more accurate microbiota profiles of positional-specific CRC.

In this study, we used the mucosal swab method to assess the mucosal microbiomes. We deemed this method suitable to examine mucosal microbes which colonized on the local intestinal mucosal surface. Due to the ruptured and bleeding epithelium of the diseased colon, some bacteria can enter tumor tissues. Biopsy samples are suitable to investigate microbes in the tumor tissues. Fecal samples contain gut microbes from almost all parts of colon and rectum, so they are commonly used to study the overall environment of the gut. Fecal samples cannot reflect the microbial profile in a certain position of the gut. However, this sampling method solely avoids the interference factor from the bowel preparation, which reduces the abundances of some gut bacteria with a low adhesion ability. Our work focused on the mucosal microbiota profiles in specific intestinal positions; therefore, the mucosal swab method was used to collect samples in situ.

The current understanding of the bacterial taxa of gut mucosal microbiota has been well improved; however, the physiological roles of the identified pathogenic bacteria in initiating and developing CRC have not yet been fully uncovered. The most notable pathogen in the gut is *Fusobacterium nucleatum*, which is an oral pathogen and plays a key role in CRC development [8],[31]–[36]. Additionally, certain other pathogenic species, including *Peptostreptococcus anaerobius*, enterotoxigenic *Bacteroides fragilis*, *pks*^+^ *Escherichia coli*, and *Eubacterium rectale*, have been demonstrated to be involved in promoting CRC by physiological experiments [9]–[11],[37]. Due to the large quantities of bacteria residing in the gut ecosystem, their taxa need to be classified at the strain level, and many potential pathogens with their toxins and virulence factors need to be characterized by experiments. There are still many important questions awaiting to be answered, especially considering the multi-dimension complexity of the ecosystem in the human gut impacted by a wide range of factors.

We found bacterial taxa with high proportions associated with positional-specific CRC in this work and summarized their previously reported potential roles (Table S1). By stringent selection, six bacterial taxa (*Beijerinckia*, *Pseudonocardia*, *Gemella*, *Fusobacterium*, *Pasteurellaceae*, and *Bacilli*) were specifically enriched at the on-tumor sites of patients with right-sided CRC, and two bacterial taxa (Left: *Lactobacillales* and *Caldisericales*; Rectum: *Methylopila* and *Parvimonas*) were identified for left-sided and rectal CRC, respectively. Besides *Fusobacterium*, species from *Gemella*, *Pasteurellaceae*, *Bacilli*, and *Parvimonas* were reported to be pathogenic or as CRC biomarkers [38]–[41]. *Lactobacillus* was reported to function as a probiotic that prevents CRC development [42]. *Beijerinckia*, *Pseudonocardia*, *Caldisericales*, and *Methylopila* were mainly isolated from soil, plants, and the environment [43]–[46]. Alternatively, *Pseudonocardia carboxydivorans* was suggested to be a potential human pathogen [44], though species from these genera have not been characterized as pathogens with overwhelming evidence. It is not excluded that these genera may play roles as opportunistic pathogens in the gut.

The bacterial taxa identified as potential biomarkers to diagnose right-sided/left-sided/rectal CRC can be classified to the following four categories: pathogen, opportunistic pathogen, probiotic, and environmental bacteria with unknown roles for humans. Species from seven genera were reported to function as human pathogens: *Plesiomonas* causes various highly infectious diseases including septicemia, meningitis, and colitis [47]; *Eubacterium rectum* induces human colitis via the Toll-like receptor 4 (TLR4) pathway [11]; *Slackia* spp. play roles in host lipid and xenobiotic metabolism, and *Slackia exigua* causes bacteremia [48]; *Granulicatella* most commonly causes endocarditis or bacteremia [49],[50]; *Sneathia amnii* functions as a pathogen which causes spondylitis, bacteremia, and chorioamnionitis [51]; *Citrobacter* induces colitis [52]; and *Vagococcus fluvialis* induces bacteremia and decubitus ulcers [53]. Three genera were reported to function as opportunistic pathogens: *Leptotrichia* typically colonizes in the oral cavity and functions as an opportunistic pathogen to cause bacteremia [54]; an *Eikenella corrodens* infection may lead to serious diseases such as periodontitis, osteromyelitis, meningitis, empyema, and endocarditis [55]; and *Finegoldia magna* induces inflammation by activating neutrophils [56],[57]. One genus is known to function as a probiotic: *Bifidobacterium longum* functions as a probiotic to suppress colorectal carcinogenesis [58],[59]. Seven genera, including *Catenibacterium*, *Labrys*, *Megasphaera*, *Acidaminococcus*, *Christensenella*, *Dickeya*, and *Turicibacter*, were reported to be isolated from plants, animals and, environmental samples [60]–[66]. The roles of species from these genera in the gut are unknown and will need efforts to be fully understood.

## 5. Conclusions

Our findings provided the gut microbiota profiles linked to right-sided, left-sided, and rectal CRC. Instead of being ubiquitously associated with tumor occurrences across the intestinal axis, unique sets of bacterial taxa were found to be enriched in positional-specific CRC. *Dickeya*, *Turicibacter*, *Plesiomonas*, *Eubacterium*, *Slackia*, *Lactobacillus*, *Leptotrichia*, *Granulicatella*, *Bifidobacterium*, and *Eikenella* can serve as potential biomarkers for right-sided CRC to be distinguished from left-sided CRC (AUC = 0.79, *P* < 0.05). *Rickettsia*, *Sneathia*, *Catenibacterium*, *Plesiomonas*, *Bifidobacterium*, *Finegoldia*, *Megasphaera*, *Lactobacillus*, and *Slackia* can serve as potential biomarkers for right-sided CRC to be distinguished from rectal CRC (AUC = 0.83, *P* < 0.05). *Sneathia*, *Labrys*, *Catenibacterium*, *Acidaminococcus*, *Citrobacter*, *Megasphaera*, *Christensenella*, and *Vagococcus* can serve as potential biomarkers to distinguish left-sided CRC from rectal CRC (AUC = 0.90, *P* < 0.05). Soon, testing the quantities of these potential biomarker sets in large-scale positional-specific CRC samples may help to develop well-designed diagnosis kits for patients with CRC in early stages.

As modern technologies such as single-cell omics develop, the complex intestinal ecosystem may be redefined and classified by compositions of cell types. Understanding the host-microbe interaction between gut resident cells and these identified bacteria in specific anatomical locations will reveal the molecular mechanisms of how each species from the gut microbiota evades host defense system and contributes to carcinogenesis. Further characterizations of the metabolic roles of these bacterial taxa in positional-specific CRC progression will help develop novel strategies for positional-specific CRC treatment and improve the prognosis outcomes.

## Use of AI tools declaration

The authors declare they have not used Artificial Intelligence (AI) tools in the creation of this article.


