# Supplementary material for: Gut mucosal microbiota profiles linked to development of positional-specific human colorectal cancer
Source: AIMS Microbiol. 2024 Sep 24;10(4):812–32. doi: 10.3934/microbiol.2024035 (PMC11609426; doi:10.3934/microbiol.2024035)
Supplement: Supplementary file 1 [file microbiol-10-04-035-s001.pdf]

Microbiology

AIMS Microbiology, 10(4): 812–832.
DOI: 10.3934/microbiol.2024035
Received: 19 March 2024
Revised: 03 September 2024
Accepted: 09 September 2024
Published: 24 September 2024

http://www.aimspress.com/journal/microbiology

Research article

Gut mucosal microbiota profiles linked to development of positional-specific human colorectal cancer

Chunze Zhang[1,6], Mingqian Ma[2], Zhenying Zhao[1], Zhiqiang Feng[2], Tianhao Chu[2], Yijia Wang[3], Jun Liu[4,*] and Xuehua Wan[5,*]

[1] Department of Colorectal Surgery, Tianjin Union Medical Center, Nankai University, Tianjin, China
[2] School of Integrative Medicine, Tianjin University of Traditional Chinese Medicine, Tianjin, China
[3] Tianjin institute of spinal surgery, Tianjin Union Medical Center, Nankai University, Tianjin, China
[4] Department of Radiology, The Fourth Central Hospital Affiliated to Nankai University, Tianjin, China
[5] TEDA Institute of Biological Sciences and Biotechnology, Nankai University, TEDA, Tianjin, China
[6] Tianjin Institute of Coloproctology, Tianjin, China

* **Correspondence:** Email: junliu_sci@163.com, xuehua.wan@nankai.edu.cn.

Supplementary

Table S1. List of bacterial taxa identified in this work.

Bacterium	Metabolism and pathogenicity	Reference
Acidaminococcus	Increased relative abundance of *Acidaminococcus* is associated with future linear growth deficits.	[1]
Actinomyces	*Actinomyces* is significantly increased in multiple polypoid adenomas and intramucosal carcinomas.	[2]
Aggregatibacter	*Aggregatibacter* is positively correlated with visceral fat, fasting plasma insulin, and HOMA-IR in non-alcoholic fatty liver disease.	[3]
Agrobacterium	Mainly known as plant pathogen. Rarely known to infect human.	[4]
Atopobium	Gram-positive, anaerobic, catalase-negative, fastidious bacteria belonging to the family *Coriobacteriaceae*. Maturation of the biofilm and coaggregation of "secondary colonizers" such as *Atopobium* spp. Five patients (18.5%) had polymicrobial bacteremia, and pathogens associated with concomitant polymicrobial infection included *Atopobium*.	[5]
Bacilli	*Bacillus cereus* human pathogen.	[6]
Beijerinckia	The genus *Beijerinckia* was initially isolated from acidic soils.	[7]
Bifidobacterium	A possible role of *Bifidobacteria* in determining distinct tumor characteristics or as an indicator of dysfunctional mucosal barrier in CRC. *Bifidobacterium longum* suppresses colorectal carcinogenesis.	[8,9]
Butyricimonas	A closely interaction between *Butyricimonas* and *Clostridium* is observed in the microbiome network in CRC samples.	[10]
Campylobacter	*Campylobacter jejuni* produces a genotoxin, cytolethal distending toxin, which has DNAse activity and causes DNA double-strand breaks and promotes colorectal tumorigenesis.	[11]
Capnocytophaga	*Capnocytophaga* causes infection in wound.	[12]
Catenibacterium	Compared to normal samples, *Catenibacterium* is only detected in CRC tumor samples.	[13]
Christensenella	The abundance of longevity related *Christensenella* species in gut microbiota increases after fasting and is inversely correlated with age as well as body mass index.	[14]
Citrobacter	*Citrobacter* is able to induce colitis.	[15]
Desulfovibrio	*Desulfovibrio* is commensal microbe colonising the mucus gel layer of the colon, metabolises the sulfate moiety of sulfated mucins, and has increased affinity to ulcerative colitis mucin.	[16]
Dialister	*Dialister* is Gram-stain-negative in healthy faecal sample, and utilizes succinate and causes repetitive bartholinitis episodes.	[17,18]
Dickeya	*Dickeya* relates to potato infection.	[19]

Continued on next page

Bacterium	Metabolism and pathogenicity	Reference
Dysgonomonas	*Dysgonomonas* is Gram-stain negative cocci. It grows optimally at 37 °C and is catalase positive but oxidase negative.	[20]
Eikenella	*Eikenella* produces lysine decarboxylase.	[21]
Eubacterium	Compared to healthy samples, abundance of *Eubacterium* decreases in patients with IBD and CRC.	[22,23]
Faecalibacterium	*Faecalibacterium* plays a major role in the regulation of gut barrier, inflammation and metabolic functions.	[24]
Finegoldia	*Finegoldia magna* is an anaerobic opportunistic pathogen.	[25,26]
Fusobacterium	*Fusobacterium* has high abundance in intestine in CRC patients, and low abundance in health intestine.	[27]
Gemella	*Gemella* is associated with CRC. The risk of CRC is increased in patients with bacteremia from *Gemella*.	[28]
Granulicatella	*Granulicatella* causes infective endocarditis and endophthalmitis.	[29,30]
Haemophilus	*Haemophilus* was depleted in the transition from stage 0 to early-stage CRC.	[31]
Klebsiella	*Klebsiella* induces pneumosepsis, produces cytotoxin, and is associated with necrotizing enterocolitis.	[32,33]
Labrys	Budding bacteria isolated from rhizosphere habitats in environments.	[34]
Lactobacillus	As a probiotic, *Lactobacillus* prevents dimethylhydrazine-induced colorectal cancers.	[35]
Leptotrichia	*Leptotrichia* is significant co-occurrence within individual tumors.	[36]
Megasphaera	A butyrate-producing bacterium.	[37]
Morganella	*Morganella* produces extended-spectrum β-lactamase, and causes wound infections after colorectal surgery.	[38]
Moryella	*Moryella* is weakly saccharolytic and produces indole, acetate, butyrate and lactate as major metabolic end products.	[39]
Neisseria	Anti-inflammatory microenvironment causes decreased *Neisseria* abundance.	[40]
Oscillospira	High *Oscillospira* abundance indicates constipation and low BMI.	[41]
Parvimonas	Bacterial biomarker of colorectal cancer.	[42]
Peptococcus	*Peptococcus magnus* human pathogen. *Peptococcus niger* is a Gram-positive, non-motile, obligatory anaerobic cocci that is a constituent of the normal human intestinal mucous membranes and umbilicus flora.	[43]
Peptoniphilus	*Peptoniphilus* is a Gram-positive anaerobic coccus mainly involved in polymicrobial infections, and is reported in a case of peritoneal infection in a patient with intestinal occlusion.	[44]
Peptostreptococcus	Bacterial biomarker of colorectal cancer.	[42]
Plesiomonas	*Plesiomonas* has several virulence factors, such as lysophospholipase, a twin-arginine translocation system and the type VI secretion effector Phospholipase A1, which relate to diarrhoeal disease.	[45]

Continued on next page

Bacterium	Metabolism and pathogenicity	Reference
Porphyromonas	*Porphyromonas gingivalis* is a keystone pathogen in periodontitis and promotes the proliferation of colorectal cancer cells by activating the MAPK/ERK signaling pathway.	[46]
Prevotella	Intestinal *Prevotella* colonization results in metabolic changes in the microbiota, which reduce IL-18 production and consequently exacerbate intestinal inflammation and potential systemic autoimmunity.	[47]
Prevotella	*Prevotella* produces butyrate and reduces abundance in CRC patients.	[48]
Pseudomonas	*Pseudomonas* produces exopolysaccharides and infection.	[49,50]
Pseudonocardia	The genus *Pseudonocardia* belongs to a group of *Actinomycetes*, and is a member of the family *Pseudonocardiacea*. The members of this genus are aerobic, Gram-positive, non-motile bacteria that are commonly found in soil, plant and environment. *Pseudonocardia carboxydivorans* may be human pathogen.	[51]
Pyramidobacter	*Pyramidobacter* produces acetic and isovaleric acids.	[52]
Rickettsia	The genus contains many human pathogens.	
Rothia	The genus *Rothia* are emerging as opportunistic pathogens associated with various infections in immunocompromised and immunocompetent individuals.	[53]
Selenomonas	*Selenomonas* is closely associated with CRC patients with hyperlipoidaemia.	[54]
Shewanella	*Shewanella* is found in water environment and in patients, and can act as theoriginator of oxacillinase in Gram-negative bacteria.	[55]
Slackia	*Slackia* is gut-associated bacteria that play roles in host lipid and xenobiotic metabolism. *Slackia* may be free living in the lumen because it has low adhesion to cells.	[56]
Sneathia	*Sneathia amnii* produces a cytotoxin, named CptA for cytopathogenic toxin, component A, which is capable of permeabilizing chorionic trophoblasts and lysing human red blood cells.	[57]
Streptococcus	*Streptococcus bovis* contributes to the development of CRC via recruiting CD11b TLR-4 cells, and should be investigated for early detection of colorectal pathology. *Streptococcus gallolyticus* subsp. *gallolyticus* (Sgg) infection has gained considerable attention for its strong association with CRC.	[58–60]
Turicibacter	*Turicibacter* is an anaerobic, Gram-positive bacterium, isolated from human feces.	[61]
Treponema	A genus of commonly found oral bacteria that are closely related to periodontitis and the etiology of implant periarthritis.The genus *Treponema* contains both pathogenic and nonpathogenic species. Human pathogens cause four treponematoses: syphilis (*T pallidum* subsp *pallidum*), yaws (*T pallidum* subsp *pertenue*), endemic syphilis (*T pallidum* subsp *endemicum*), and pinta (*T carateum*). Nonpathogenic treponemes may be part of the normal flora of the intestinal tract, the oral cavity, or the genital tract. Some of the oral treponemes have been associated with gingivitis and periodontal disease.	[62]
Vagococcus	The *Vagococcus* is a relatively recently recognized genus of Gram-positive, catalase-negative cocci.	[63]
Veillonella	A causative pathogen of bloodstream and decubitus ulcer infection.	[64]

Reference

1. Gough EK, Stephens DA, Moodie EE, et al. (2015) Linear growth faltering in infants is associated with *Acidaminococcus* sp. and community-level changes in the gut microbiota. *Microbiome* 3: 24. https://doi.org/10.1186/s40168-015-0089-2
2. Yachida S, Mizutani S, Shiroma H, et al. (2019) Metagenomic and metabolomic analyses reveal distinct stage-specific phenotypes of the gut microbiota in colorectal cancer. *Nat Med* 25: 968–976. https://doi.org/10.1038/s41591-019-0458-7
3. Komazaki R, Katagiri S, Takahashi H, et al. (2017) Periodontal pathogenic bacteria, *Aggregatibacter actinomycetemcomitans* affect non-alcoholic fatty liver disease by altering gut microbiota and glucose metabolism. *Sci Rep* 7: 13950. https://doi.org/10.1038/s41598-017-14260-9
4. Chalandon Y, Roscoe D, Nantel S (2000) *Agrobacterium* yellow group: Bacteremia and possible septic arthritis following peripheral blood stem cell transplantation. *Bone Marrow Transplant* 26: 101–104. https://doi.org/10.1038/sj.bmt.1702470
5. Watanabe T, Hara Y, Yoshimi Y, et al. (2020) Clinical characteristics of bloodstream infection by *Parvimonas micra*: Retrospective case series and literature review. *BMC Infect Dis* 20: 578. https://doi.org/10.1186/s12879-020-05305-y
6. Bottone EJ (2010) *Bacillus cereus*, a volatile human pathogen. *Clin Microbiol Rev* 23: 382–398. https://doi.org/10.1128/cmr.00073-09
7. Gamit HA, Amaresan N (2022) Isolation and identification of *Beijerinckia*, In: *Practical Handbook on Agricultural Microbiology*, New York: Springer, 119–125. https://doi.org/10.1007/978-1-0716-1724-3_15
8. Kosumi K, Hamada T, Koh H, et al. (2018) The amount of *Bifidobacterium* genus in colorectal carcinoma tissue in relation to tumor characteristics and clinical outcome. *Am J Pathol* 188: 2839–2852. https://doi.org/10.1016/j.ajpath.2018.08.015
9. Fahmy CA, Gamal-Eldeen AM, El-Hussieny EA, et al. (2019) *Bifidobacterium longum* suppresses murine colorectal cancer through the modulation of oncomiRs and tumor suppressor miRNAs. *Nutr Cancer* 71: 688–700. https://doi.org/10.1080/01635581.2019.1577984
10. Zhang Q, Zhao H, Wu D, et al. (2020) A comprehensive analysis of the microbiota composition and gene expression in colorectal cancer. *BMC Microbiol* 20: 308. https://doi.org/10.1186/s12866-020-01938-w
11. He Z, Gharaibeh RZ, Newsome RC, et al. (2019) *Campylobacter jejuni* promotes colorectal tumorigenesis through the action of cytolethal distending toxin. *Gut* 68: 289–300. https://doi.org/10.1136/gutjnl-2018-317200
12. Markewitz RDH, Graf T (2020) *Capnocytophaga canimorsus* infection. *N Engl J Med* 383: 1167. https://doi.org/10.1056/NEJMicm1916407
13. Zhang Q, Zhao H, Wu D, et al. (2020) A comprehensive analysis of the microbiota composition and gene expression in colorectal cancer. *BMC Microbiol* 20: 308. https://doi.org/10.1186/s12866-020-01938-w
14. Lilja S, Stoll C, Krammer U, et al. (2021) Five days periodic fasting elevates levels of longevity related *Christensenella* and Sirtuin expression in humans. *Int J Mol Sci* 22: 2331. https://doi.org/10.3390/ijms22052331

15. Vong L, Pinnell LJ, Määttänen P, et al. (2015) Selective enrichment of commensal gut bacteria protects against Citrobacter rodentium-induced colitis. *Am J Physiol Gastrointest Liver Physiol* 309: G181–G192. https://doi.org/10.1152/ajpgi.00053.2015
16. Earley H, Lennon G, Balfe A, et al. (2015) A preliminary study examining the binding capacity of *Akkermansia muciniphila* and *Desulfovibrio* spp., to colonic mucin in health and ulcerative colitis. *PLoS One* 10: e0135280. https://doi.org/10.1371/journal.pone.0135280
17. Sakamoto M, Ikeyama N, Toyoda A, et al. (2020) *Dialister hominis* sp. nov., isolated from human faeces. *Int J Syst Evol Microbiol* 70: 589–595. https://doi.org/10.1099/ijsem.0.003797
18. Cobo F, Rodríguez-Granger J, Sampedro A, et al. (2018) Bartholin's abscess due to *Dialister micraerophilus* in a woman presenting with repetitive bartholinitis episodes. *Med Mal Infect* 48: 225–226. https://doi.org/10.1016/j.medmal.2017.12.008
19. Ge T, Ekbataniamiri F, Johnson SB, et al. (2021) Interaction between *Dickeya dianthicola* and *Pectobacterium parmentieri* in potato infection under field conditions. *Microorganisms* 9: 2. https://doi.org/10.3390/microorganisms9020316
20. Bilen M, Fonkou MDM, Dubourg G, et al. (2019) *Dysgonomonas massiliensis* sp. nov., a new species isolated from the human gut and its taxonogenomic description. *Antonie Van Leeuwenhoek* 112: 935–945. https://doi.org/10.1007/s10482-019-01227-1
21. Levine M, Collins LM, Lohinai Z (2021) Zinc chloride inhibits lysine decarboxylase production from *Eikenella corrodens in vitro* and its therapeutic implications. *J Dent* 104: 103533. https://doi.org/10.1016/j.jdent.2020.103533
22. Zhang S, Wang S, Miao C (2017) Influence of microbiota on intestinal immune system in ulcerative colitis and its intervention. *Front Immunol* 8: 1674. https://doi.org/10.3389/fimmu.2017.01674
23. Wang Y, Zhang C, Hou S, et al. (2020) Analyses of potential driver and passenger bacteria in human colorectal cancer. *Cancer Manage Res* 12: 11553–11561. https://doi.org/10.2147/CMAR.S275316
24. Moosavi SM, Akhavan Sepahi A, Mousavi SF, et al. (2020) The effect of *Faecalibacterium prausnitzii* and its extracellular vesicles on the permeability of intestinal epithelial cells and expression of PPARs and ANGPTL4 in the Caco-2 cell culture model. *J Diabetes Metab Disord* 19: 1061–1069. https://doi.org/10.1007/s40200-020-00605-1
25. Brüggemann H, Jensen A, Nazipi S, et al. (2018) Pan-genome analysis of the genus *Finegoldia* identifies two distinct clades, strain-specific heterogeneity, and putative virulence factors. *Sci Rep* 8: 266. https://doi.org/10.1038/s41598-017-18661-8
26. de Moreuil C, Héry-Arnaud G, David CH, et al. (2015) *Finegoldia magna*, not a well-known infectious agent of bacteriemic post-sternotomy mediastinitis. *Anaerobe* 32: 32–33. https://doi.org/10.1016/j.anaerobe.2014.11.012
27. Abed J, Emgård JEM, Zamir G, et al. (2016) Fap2 mediates *Fusobacterium nucleatum* colorectal adenocarcinoma enrichment by binding to tumor-expressed Gal-GalNAc. *Cell Host Microbe* 20: 215–225. https://doi.org/10.1016/j.chom.2016.07.006
28. Kwong TNY, Wang X, Nakatsu G, et al. (2018) Association between bacteremia from specific microbes and subsequent diagnosis of colorectal cancer. *Gastroenterology* 155: 383–390.e8. https://doi.org/10.1053/j.gastro.2018.04.028
29. Alkandari SA, Bhardwaj RG, Ellepola A, et al. (2020) Proteomics of extracellular vesicles produced by *Granulicatella adiacens*, which causes infective endocarditis. *PLoS One* 15: e0227657. https://doi.org/10.1371/journal.pone.0227657

30. Al-Lozi A, Cai S, Chen X, et al. (2021) *Granulicatella adiacens* as an unusual cause of microbial keratitis and endophthalmitis: a case series and literature review. *Ocul Immunol Inflamm* 30: 1181–1185. https://doi.org/10.1080/09273948.2020.1860233
31. Yang T, Lee W, Tu S, et al. (2019) Enterotype-based analysis of gut microbiota along the conventional adenoma-carcinoma colorectal cancer pathway. *Sci Rep* 9: 10923. https://doi.org/10.1038/s41598-019-45588-z
32. Wu T, Xu F, Su C, et al. (2020) Alterations in the gut microbiome and cecal metabolome during *Klebsiella pneumoniae*-induced pneumosepsis. *Front Immunol* 11: 1331. https://doi.org/10.3389/fimmu.2020.01331
33. Paveglio S, Ledala N, Rezaul K, et al. (2020) Cytotoxin-producing *Klebsiella oxytoca* in the preterm gut and its association with necrotizing enterocolitis. *Emerg Microbes Infect* 9: 1321–1329. https://doi.org/10.1080/22221751.2020.1773743
34. Islam MS, Kawasaki H, Nakagawa Y, et al. (2007) *Labrys okinawensis* sp. nov. and *Labrys miyagiensis* sp. nov., budding bacteria isolated from rhizosphere habitats in Japan, and emended descriptions of the genus *Labrys* and *Labrys monachus*. *Int J Syst Evol Microbiol* 57: 552–557. https://doi.org/10.1099/ijs.0.64239-0
35. Dong Y, Zhu J, Zhang M, et al. (2020) Probiotic *Lactobacillus salivarius* Ren prevent dimethylhydrazine-induced colorectal cancer through protein kinase B inhibition. *Appl Microbiol Biotechnol* 104: 7377–7389. https://doi.org/10.1007/s00253-020-10775-w
36. Warren RL, Freeman DJ, Pleasance S, et al. (2013) Co-occurrence of anaerobic bacteria in colorectal carcinomas. *Microbiome* 1: 16. https://doi.org/10.1186/2049-2618-1-16
37. Maki JJ, Looft T (2018) *Megasphaera stantonii* sp. nov., a butyrate-producing bacterium isolated from the cecum of a healthy chicken. *Int J Syst Evol Microbiol* 68: 3409–3415. https://doi.org/10.1099/ijsem.0.002991
38. Kalakouti E, Simillis C, Pellino G, et al. (2017) Characteristics of surgical site infection following colorectal surgery in a tertiary center: Extended-spectrum β-lactamase-producing bacteria culprits in disease. *Wounds* 30: 108–113.
39. Carlier JP, K'ouas G, Han XY (2007) *Moryella indoligenes* gen. nov., sp. nov., an anaerobic bacterium isolated from clinical specimens. *Int J Syst Evol Microbiol* 57: 725–729. https://doi.org/10.1099/ijs.0.64705-0
40. Rodríguez-García C, Sánchez-Quesada C, Algarra I, et al. (2020) The high-fat diet based on extra-virgin olive oil causes dysbiosis linked to colorectal cancer prevention. *Nutrients* 12: 1705. https://doi.org/10.3390/nu12061705
41. Chen Y, Zheng H, Zhang G, et al. (2020) High *Oscillospira* abundance indicates constipation and low BMI in the Guangdong gut microbiome project. *Sci Rep* 10: 9364. https://doi.org/10.1038/s41598-020-66369-z
42. Osman MA, Neoh HM, Ab Mutalib NS, et al. (2021) *Parvimonas micra*, *Peptostreptococcus stomatis*, *Fusobacterium nucleatum* and *Akkermansia muciniphila* as a four-bacteria biomarker panel of colorectal cancer. *Sci Rep* 11: 2925. https://doi.org/10.1038/s41598-021-82465-0
43. Bourgault AM, Rosenblatt JE, Fitzgerald RH (1980) *Peptococcus magnus*: A significant human pathogen. *Ann Intern Med* 93: 244–248. https://doi.org/10.7326/0003-4819-93-2-244
44. Cobo F, Rodríguez-Granger J, Sampedro A, et al. (2017) Peritoneal infection due to *Peptoniphilus harei* in a patient with intestinal occlusion. *Anaerobe* 44: 126–127. https://doi.org/10.1016/j.anaerobe.2017.03.009

45. Edwards MS, McLaughlin RW, Li J, et al. (2019) Putative virulence factors of *Plesiomonas shigelloides*. *Antonie Van Leeuwenhoek* 112: 1815–1826. https://doi.org/10.1007/s10482-019-01303-6
46. Mu W, Jia Y, Chen X, et al. (2020) Intracellular *Porphyromonas gingivalis* promotes the proliferation of colorectal cancer cells via the MAPK/ERK signaling pathway. *Front Cell Infect Microbiol* 10: 584798. https://doi.org/10.3389/fcimb.2020.584798
47. Iljazovic A, Roy U, Gálvez EJC, et al. (2021) Perturbation of the gut microbiome by *Prevotella* spp. enhances host susceptibility to mucosal inflammation. *Mucosal Immunol* 14: 113–124. https://doi.org/10.1038/s41385-020-0296-4
48. Huang CC, Shen MH, Chen SK, et al. (2020) Gut butyrate-producing organisms correlate to placenta specific 8 protein: Importance to colorectal cancer progression. *J Adv Res* 22: 7–20. https://doi.org/10.1016/j.jare.2019.11.005
49. Jennings LK, Dreifus JE, Reichhardt C, et al. (2021) *Pseudomonas aeruginosa* aggregates in cystic fibrosis sputum produce exopolysaccharides that likely impede current therapies. *Cell Rep* 34: 108782. https://doi.org/10.1016/j.celrep.2021.108782
50. Askarian F, Uchiyama S, Masson H, et al. (2021) The lytic polysaccharide monooxygenase CbpD promotes *Pseudomonas aeruginosa* virulence in systemic infection. *Nat Commun* 12: 1230. https://doi.org/10.1038/s41467-021-21473-0
51. Riahi HS, Heidarieh P, Fatahi-Bafghi M (2021) Genus Pseudonocardia: What we know about its biological properties, abilities and current application in biotechnology. *J Appl Microbiol* 132: 759–1556. https://doi.org/10.1111/jam.15271
52. Downes J, Vartoukian SR, Dewhirst FE, et al. (2009) *Pyramidobacter piscolens* gen. nov., sp. nov., a member of the phylum '*Synergistetes*' isolated from the human oral cavity. *Int J Syst Evol Microbiol* 59: 972–980. https://doi.org/10.1099/ijs.0.000364-0
53. Fatahi-Bafghi M (2021) Characterization of the *Rothia* spp. and their role in human clinical infections. *Infect Genet Evol* 93: 104877. https://doi.org/10.1016/j.meegid.2021.104877
54. Han S, Pan Y, Yang X, et al. (2019) Intestinal microorganisms involved in colorectal cancer complicated with dyslipidosis. *Cancer Biol Ther* 20: 81–89. https://doi.org/10.1080/15384047.2018.1507255
55. Leangapichart T, Hadjadj L, Gautret P, et al. (2021) Comparative genomics of two *Shewanella xiamenensis* strains isolated from a pilgrim before and during travels to the Hajj. *Gut Pathog* 13: 9. https://doi.org/10.1186/s13099-021-00404-w
56. Cho GS, Ritzmann F, Eckstein M, et al. (2016) Quantification of *Slackia* and *Eggerthella* spp. in human feces and adhesion of representatives strains to Caco-2 cells. *Front Microbiol* 7: 658. https://doi.org/10.3389/fmicb.2016.00658
57. Gentile GL, Rupert AS, Carrasco LI, et al. (2020) Identification of a cytopathogenic toxin from *Sneathia amnii*. *J Bacteriol* 202: e00162–e00220. https://doi.org/10.1128/jb.00162-20
58. Deng Q, Wang C, Yu K, et al. (2020) Streptococcus bovis contributes to the development of colorectal cancer via recruiting $CD11b^{+}TLR\text{-}4^{+}$ cells. *Med Sci Monit* 26: e921886. https://doi.org/10.12659/MSM.921886
59. Ng SC, Wong HK, So CK, et al. (2019) *Streptococcus bovis* bacteraemia should be investigated for early detection of colorectal pathology. *Hong Kong Med J* 25: 414. https://doi.org/10.12809/hkmj198135

60. Lin Q, Ye X, Yang B, et al. (2019) Real-time fluorescence loop-mediated isothermal amplification assay for rapid and sensitive detection of *Streptococcus gallolyticus* subsp. *gallolyticus* associated with colorectal cancer. *Anal Bioanal Chem* 411: 6877–6887. https://doi.org/10.1007/s00216-019-02059-8
61. Cuív PÓ, Klaassens ES, Durkin AS, et al. (2011) Draft genome sequence of *Turicibacter sanguinis* PC909, isolated from human feces. *J Bacteriol* 193: 1288–1289. https://doi.org/10.1128/JB.01328-10
62. Radolf JD Treponema, *Medical Microbiology*, 4 Eds., Galveston: University of Texas Medical Branch at Galveston. Available from: https://www.ncbi.nlm.nih.gov/books/NBK7716/.
63. Matsuo T, Mori N, Kawai F, et al. (2021) *Vagococcus fluvialis* as a causative pathogen of bloodstream and decubitus ulcer infection: case report and systematic review of the literature. *J Infect Chemother* 27: 359–363. https://doi.org/10.1016/j.jiac.2020.09.019
64. Sheng Q, Du H, Cheng X, et al. (2019) Characteristics of fecal gut microbiota in patients with colorectal cancer at different stages and different sites. *Oncol Lett* 18: 4834–4844. https://doi.org/10.3892/ol.2019.10841

AIMS Press

© 2024 the Author(s), licensee AIMS Press. This is an open access article distributed under the terms of the Creative Commons Attribution License (http://creativecommons.org/licenses/by/4.0)
